# H3K4 tri-methylation breadth at transcription start sites impacts the transcriptome of systemic lupus erythematosus

**DOI:** 10.1186/s13148-016-0179-4

**Published:** 2016-02-02

**Authors:** Zhe Zhang, Lihua Shi, Noor Dawany, Judith Kelsen, Michelle A. Petri, Kathleen E. Sullivan

**Affiliations:** Department of Biomedical and Health Informatics, The Children’s Hospital of Philadelphia, Philadelphia, PA 19104 USA; Division of Allergy and Immunology, The Children’s Hospital of Philadelphia, Philadelphia, PA 19104 USA; Division of Gastroenterology, The Children’s Hospital of Philadelphia, Philadelphia, PA 19104 USA; Division of Rheumatology, Johns Hopkins University School of Medicine, Baltimore, MD 21287 USA

**Keywords:** Systemic lupus erythematosus, H3K4me3, Epigenome, Integrative analysis, Pattern recognition

## Abstract

**Background:**

The autoimmune disease systemic lupus erythematosus (SLE) has a modified epigenome with modified tri-methylation of histone H3 lysine 4 (H3K4me3) at specific loci across the genome. H3K4me3 is a canonical chromatin mark of active transcription. Recent studies have suggested that H3K4me3 breadth has an important regulatory role in cell identity. This project examined H3K4me3 breadth at transcription start sites (TSS) in primary monocytes and its association with differential gene transcription in SLE.

**Results:**

Integrative analysis was applied to chromatin immunoprecipitation sequencing (ChIP-seq) and RNA sequencing (RNA-seq) data generated from primary monocytes as well as genomic data available in public repositories. Four distinctive H3K4me3 patterns of ChIP-seq peaks were identified at 8399 TSSs. Narrow peaks were highly enriched with genes related to housekeeping functions. The broader peaks with extended H3K4me3 immediately upstream and/or downstream of TSS were associated with immune response genes. Many TSSs had downstream H3K4me3 extended to ~650 bp, where the transition of H3K4me3 to H3K36me3, a transcriptional elongation mark, is often found. The H3K4me3 pattern was strongly associated with transcription in SLE. Genes with narrow peaks were less likely (OR = 0.14, *p* = 2 × 10^−4^) while genes with extended downstream H3K4me3 were more likely (OR = 2.37, *p* = 1 × 10^−11^) to be overexpressed in SLE. Of the genes significantly overexpressed in SLE, 78.8 % had increased downstream H3K4me3 while only 47.1 % had increased upstream H3K4me3. Gene transcription sensitively and consistently responded to H3K4me3 change downstream of TSSs. Every 1 % increase of H3K4me3 in this region leads to ~1.5 % average increase of transcription.

**Conclusions:**

We identified the immediate TSS downstream nucleosome as a crucial regulator responsible for transcription changes in SLE. This study applied a unique method to study the effect of H3K4me3 breadth on diseases and revealed new insights about epigenetic modifications in SLE, which could lead to novel treatments.

**Electronic supplementary material:**

The online version of this article (doi:10.1186/s13148-016-0179-4) contains supplementary material, which is available to authorized users.

## Background

Tri-methylation of histone H3 lysine 4 (H3K4me3) is a major chromatin mark regulating gene transcription [1]. It is mostly found around transcription start sites (TSS) and strongly associated with active transcription [2, 3]. Active chromatin marks such as H3K4me3 are typically restricted to narrow regions over specific functional genomic motifs while repressive marks, such as H3K27me3, are often deposited over broader genomic regions [4]. However, very broad peaks of H3K4me3 were recently identified in many cell types as marks that predicted cell identity [5]. These broad peaks spanned up to 60 kb and highly differed between cell types [6, 7]. It was further shown that the breadth of H3K4me3 regions was positively correlated with transcriptional consistency [5]. The relationship between RNA polymerase pausing and transcriptional consistency may explain the regulatory role of these broad regions of H3K4me3. However, a mechanism linking them definitively has not yet been identified. Control of transcriptional noise may be permissive for cell fate decisions, and therefore, tight regulation of transcriptional consistency may be required for full commitment to a phenotype [8]. The goal of this study was to identify distinctive patterns of H3K4me3 peak breadth within a narrower region around TSSs and determine if H3K4me3 breadth specifically at TSSs represented an independent variable of transcription regulation, a topic not previously investigated in diseases.

Systemic lupus erythematosus (SLE) is a systemic autoimmune disease affecting nearly all types of hematopoietic cells. This polygenic disorder has a complex etiopathogenesis that involves the production of type I interferon, production of autoantibody, and T cell anomaly [9]. Clinical manifestations of SLE include arthritis, nephritis, and dermatitis. We had previously noted a markedly altered epigenome in SLE [10–13]. In this study, we utilized our previous chromatin immunoprecipitation sequencing (ChIP-seq) and RNA sequencing (RNA-seq) data sets to re-analyze chromatin changes from the perspective of peak breadth rather than peak height. After finding that substantial differences in peak breadth were strongly associated with transcription regulation, we were able to identify that the nucleosome downstream of the transcription start site is more directly associated with differential transcription than the upstream nucleosome in SLE.

## Results and discussion

### Classification of TSSs by their H3K4me3 breadth in primary monocytes

H3K4me3 breadth has been identified as a regulator of cell identity and transcriptional consistency in differentiating cells [5]. We studied the H3K4me3 breadth and the pattern of H3K4me3 marks specifically around 27,588 unique TSSs in human primary monocytes to understand whether it was an independent variable of transcription regulation. The average H3K4me3, from 250 bp upstream to 250 bp downstream of TSSs, followed a bimodal distribution (Additional file 1: Figure S1A). The left peak corresponds to random background from sites without H3K4me3, whereas the right peak corresponds to the range of H3K4me3 levels at the other sites. We selected 14,217 TSSs with significantly higher H3K4me3 than the background for further analyses.

**Fig. 1:**
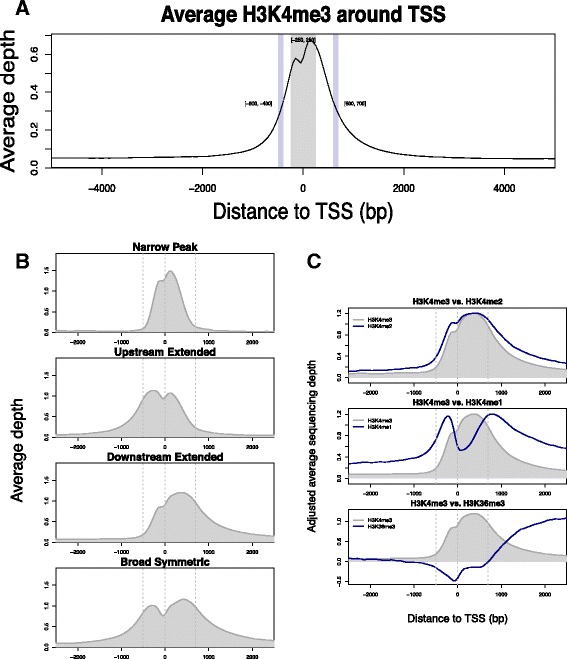
H3K4me3 at TSS. **a** The average H3K4me3 around TSS reduces by 50 % at ~400–500 bp upstream and ~600–700 bp downstream. This plot was based on the normalized average sequencing depth of six control samples. **b** Four distinctive patterns of TSS H3K4me3 were defined: narrow peak, upstream extended downstream extended, and broad symmetric. The same control samples were used for this plot. **c** Complementary pattern of other histone modifications to the downstream extended pattern of H3K4me3. Data of the other histone modifications in CD14+ monocyte were provided by the ENCODE project

In accordance with the described canonical H3K4me3 pattern, our samples showed that H3K4me3 was clearly enriched at TSS (Fig. 1a). A closer look at individual sites suggested that the shape of H3K4me3 peaks were neither necessarily symmetric nor comparable across all genes. Sites having H3K4me3 that dropped immediately from TSS on both sides corresponded to narrow H3K4me3 peaks. Conversely, H3K4me3 that dropped slowly from TSS corresponded to broader H3K4me3 peaks. To identify different patterns, we first calculated the upstream-TSS and downstream-TSS differences of H3K4me3 at all TSS sites, which both had bimodal distribution (Additional file 1: Figure S1B). We were then able to classify 8399 (~60 %) TSSs with high confidence into four distinctive H3K4me3 patterns: (1) narrow peak, sites with narrow H3K4me3 limited to the TSS; (2) upstream extended, sites with extended H3K4me3 upstream of TSSs only; (3) downstream extended, sites with extended H3K4me3 downstream of TSSs only; and (4) broad symmetric, sites with extended H3K4me3 at both ends (Fig. 1b).

We used ENCODE ChIP-seq data generated from CD14+ monocyte to get a detailed histone landscape related to H3K4me3 breadth. We first repeated the classification of TSSs using the ENCODE H3K4me3 data. The two classifications agreed on 87 % of classified TSSs (kappa = 0.8, *p* < 2.2 × 10^−16^). None of the narrow peaks classified by one data set were re-classified as any of the broad peak patterns by the other data set, suggesting that narrow H3K4me3 at TSS is a stable feature. Furthermore, ENCODE data showed that other histone modifications complemented to the patterns of H3K4me breadth (Additional file 2: Figure S2). For example, H3K4me2 has almost the same pattern as H3K4me3, H3K4me1 tends to locate next to the outer edge of H3K4me3, and H3K36me3 often increases immediately after downstream extended H3K4me3 (Fig. 1c).

### Association between H3K4me3 breadth at TSS and gene function

We ran overrepresentation analysis to associate predefined gene sets with each of the four H3K4me3 patterns using the hypergeometric test for statistical significance and all 14,217 TSSs with H3K4me3 as background. The narrow peak H3K4me3 pattern was highly significantly overrepresented by genes related to housekeeping functions such as mitochondria, ribosome, and cellular membrane genes. The 3045 TSSs with the upstream extended pattern of H3K4me3 were enriched with the target genes of many transcription factors (TFs), such as SP1 and CREB. The 2789 TSSs with the downstream extended pattern of H3K4me3 were highly enriched with immune response genes. Lastly, the 1752 TSSs with the broad symmetric peak pattern of H3K4me3 were enriched with TF target genes and genes regulating DNA binding (Table 1). Noticeably, the downstream extended pattern was closely aligned with genes related to monocyte intrinsic functions, and all three of the non-narrow peak patterns were associated with SLE-related genes. The downstream extended pattern was also associated with interferon responses, a central feature of SLE [14–16]. Therefore, the H3K4me3 patterns are not simply variations on a theme but definite specific subsets of functionally related genes.

**Table 1:** Selected overrepresented gene sets associated with each H3K4me3 pattern

H3K4me3	Source	Name	*N*	Odds ratio	*p* value	FDR
Narrow peak	BioSystems	tRNA metabolic process	28	4.001	1.68E–18	1.29E–14
Narrow peak	BioSystems	Oxidative phosphorylation	11	4.494	1.68E–09	4.39E–07
Narrow peak	KEGG	Exon junction complex (EJC)	4	7.094	8.03E–06	4.46E–04
Narrow peak	BioSystems	RNA polymerase II core binding	4	6.305	1.66E–05	7.59E–04
Upstream extended	MSigDb	FOXM1	38	2.304	2.39E–10	3.68E–06
Upstream extended	MSigDb	PAX4	42	2.003	7.99E–09	3.73E–05
Upstream extended	MSigDb	MYOD	31	2.225	2.09E–08	5.36E–05
Upstream extended	KEGG	Systemic lupus erythematosus	22	1.572	1.07E–03	5.61E–02
Downstream extended	KEGG	Hematopoietic cell lineage	27	14.792	1.24E–30	1.85E–28
Downstream extended	KEGG	*Staphylococcus aureus* infection	13	9.427	1.98E–13	3.90E–12
Downstream extended	MSigDb	Systemic lupus erythematosus	12	5.216	2.53E–09	2.50E–08
Downstream extended	BioSystems	Response to interferon-alpha	8	8.681	1.23E–08	1.09E–07
Broad symmetric	BioSystems	RORA activates gene expression	11	14.111	1.02E–15	3.65E–14
Broad symmetric	BioSystems	DNA binding, bending	8	12.285	2.28E–11	3.97E–10
Broad symmetric	MSigDb	HMX1	10	6.987	9.52E–11	1.48E–09
Broad symmetric	KEGG	Systemic lupus erythematosus	18	2.198	4.11E–06	2.44E–05

Source: The original database that defines the gene set, *Name* original gene set name, *N* number of genes belong to both the H3K4me3 pattern and the gene set, *Odds ratio* relative enrichment, *p value* hypergeometric test *p* value, *FDR* false discovery rate by Benjamini/Hochberg method

### Association of H3K4me3 breadth at TSS with gene transcription

**Fig. 2:**
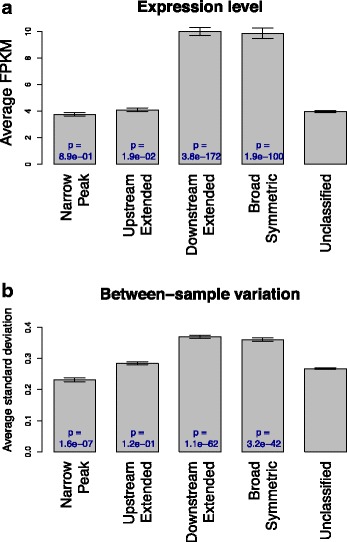
Transcription and H3K4me3 pattern. Control samples were used to examine the relationship between gene transcription and H3K4me3 patterns. **a** Genes with the downstream extended H3K4me3 pattern had higher level of transcription. **b** Genes with the downstream extended H3K4me3 pattern had higher between sample variance of transcription. The dependence of between-sample variance on transcription level was removed using the Loess local fitting method, as genes having higher transcription tend to have higher variance too. *Error bars* represent standard errors, and *p* values were calculated by comparing each pattern to unclassified group using Wilcoxon rank-sum test

We compared transcription level and between-sample variance of genes associated with the four H3K4me3 patterns, using the RNA-seq data derived from the same control samples. Comparing to sites not classified into any of the four patterns, TSSs classified into any of the non-narrow H3K4me3 patterns were associated with higher transcription (Fig. 2a). The downstream extended pattern was most strongly associated with high transcription (+151 % on average). The downstream extended pattern of H3K4me3 was also associated with higher between-sample variance of gene transcription even after the dependence of variance on transcription level was removed by fitting a nonlinear regression model (Fig. 2b).

**Fig. 3:**
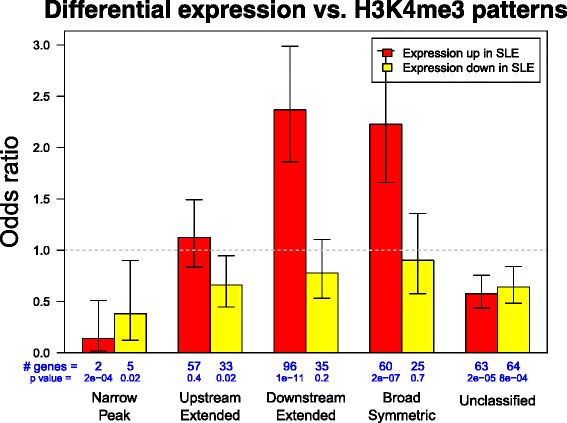
Differential transcription in SLE and H3K4me3 pattern. Genes with certain H3K4m43 patterns at their TSS were significantly less or more likely to have increased transcription in SLE. Fisher’s exact test was applied to each H3K4me3 pattern and genes with significantly decreased and increased transcription in SLE to calculate odds ratios and error bars

From our previous RNA-seq data of both SLE and control samples, we identified 1122 and 775 genes having, respectively, increased and decreased transcription in SLE patients (*p* < 0.01) [11]. There is a significant association between baseline H3K4me3 patterns at TSSs and the likelihood of increased transcription in SLE (Fig. 3). Genes having increased transcription were less likely to have the narrow peak pattern (OR = 0.14, *p* = 2 × 10^−4^, Fisher’s exact test) and more likely to have with the downstream extended pattern (OR = 2.37, *p* = 1 × 10^−11^). These results further confirmed the housekeeping functions of genes having narrow peak H3K4me3 and suggested that H3K4me3 downstream of TSSs was critical to increased transcription of genes regulating immune responses and SLE disease state. On the other hand, no significant association was found between decreased gene transcription and H3K4me3 patterns at TSSs.

### The change of H3K4me3 breadth in SLE and its impact on gene transcription

The 8399 TSSs classified into the four H3K4me3 patterns by the control data were re-classified using the data from six SLE patients. Of those TSSs sites, 94.8 % were classified into the same group (kappa = 0.93, *p* < 2.2 × 10^−16^), suggesting that H3K4me3 breadth is a stable feature in primary monocytes even in disease state. Among the 907 TSSs that switched patterns, none of them switched from the narrow peak pattern in controls to any of the non-narrow patterns in patients (Table 2). Pattern switching was not strongly associated with baseline H3K4me3 level, transcription level, or transcription changes in SLE.

**Table 2:** Agreement between controls and SLE patients on classification of TSSs based on their H3K4me3 patterns

	Narrow peak	Upstream extended	Downstream extended	Broad symmetric
Narrow peak	647	0	0	0
Upstream extended	0	2630	6	116
Downstream extended	0	2	2484	97
Broad symmetric	0	74	98	1529

Rows are numbers of TSSs classified into each H3K4me3 pattern according to control samples. Columns are numbers of TSSs classified by SLE samples

**Fig. 4:**
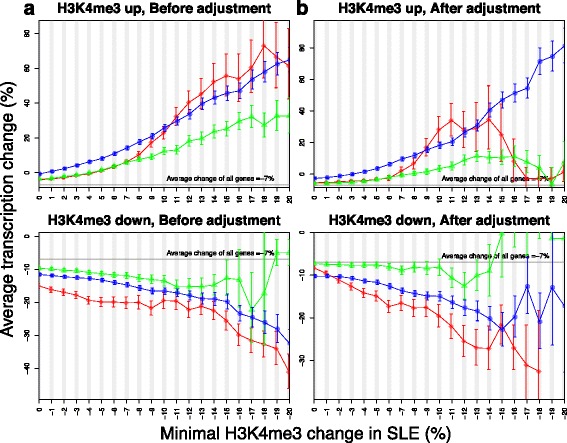
The effect of H3K4me3 change on differential transcription in SLE. **a** Each plot shows the average change of transcription in SLE in response to 1 % of H3K4me3 increase or decrease at three neighboring regions. **b** Such association was re-evaluated after removing the dependence of H3K4me3 at these regions on each other. *Red diamonds* represent the TSS, *green triangles* represent the upstream region, and the *blue circles* represent the downstream region. *Error bars* represent standard errors

We previously reported that increase of H3K4me3 peak height at TSSs was associated with higher transcription of the corresponding genes in SLE [17]. To better define the impact of location-specific H3K4me3 change on transcription, we divided the site around each TSS into three regions: TSS, upstream (−400 to −300 bp), and downstream (+600 to +700 bp). H3K4me3 and transcription changes were positively correlated at all three regions (Fig. 4a). The correlation was stronger at TSS and downstream than at upstream. Transcription change was very sensitive to H3K4me3 change at the downstream region. From 1 to 10 %, every 1 % increase in H3K4me3 at this region led to a 1.5 % increase of transcription on average. On the other hand, 5 % or more increase of H3K4me3 at TSS itself was necessary to have a notable impact on average transcription. Therefore, the H3K4me3 at the downstream region had a more direct effect on gene transcription.

Since H3K4me3 of adjacent regions are closely correlated with each other, we re-analyzed the correlation between H3K4me3 and transcription changes at these three regions after removing their dependence on each other (Fig. 4b). The positive correlation between upstream H3K4me3 and transcription changes was diminished, suggesting that the previously observed association was an artifact and change of upstream H3K4me3 indirectly affected transcription through modifying the chromatin accessibility of other transcription regulators. On the other hand, the association between downstream H3K4me3 and transcription remained, suggesting that change in downstream H3K4me3 directly affected transcription probably through facilitating transcription elongation.

**Fig. 5:**
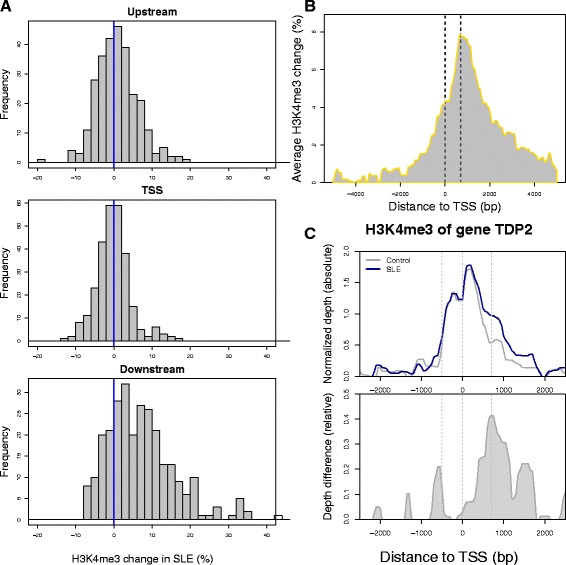
H3K4me3 patterns of increased gene transcription in SLE. **a** The distributions of H3K4me3 changes at genes with increased transcription were different among three nearby regions after their dependence on each other was removed. Each bar defines the number of genes and the *x*-axis displays the magnitude of H3K4me3 change. **b** The TSS H3K4me3 of genes with increased transcription in SLE had the most increase at ~700 bp. The *dotted lines* represent TSS and the peak summit. **c** Relative change of H3K4me3 at upstream and downstream of TSS is often shadowed by higher level of H3K4me3 at TSS when sequencing depth of control and patient samples was plotted separately (*upper panel*), and becomes more prominent when the relative depth difference is plotted (*lower panel*). TDP2 encodes a protein associated with major regulators of immune response, such as CD40, TNF, and TRAFs. Its transcription was significantly increased by 96 % in SLE (*p* = 0.004). We encourage readers to use our online tool (see “Methods” section) to make similar plots of their genes of interest

Of the 1122 genes having significantly higher transcription in SLE, 78.8 % also had increased H3K4me3 at the downstream regions, while the percentages were 55.0 and 47.1 % for TSSs and upstream regions, respectively (Fig. 5a). Indeed, the average H3K4me3 change of these genes peaked at approximately 700 bp downstream of their TSSs (Fig. 5b). Since the upstream and downstream changes were not dramatic in most cases, they were often overlooked due to the high TSS H3K4me3 peaks in landscape plots (Fig. 5c). These data suggest that the preferable way to visualize H3K4me3 changes at different regions is to plot the relative patient-control difference instead of absolute sequencing depth.

### Protein-binding motifs associated with H3K4me3 breadth

**Fig. 6:**
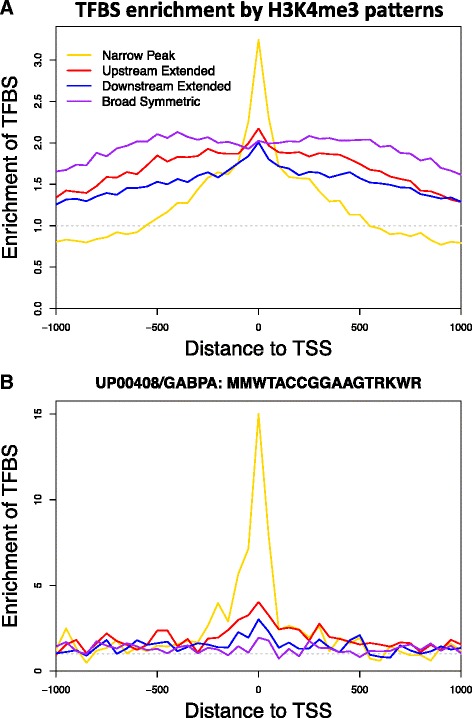
TFBS enrichment of H3K4me3 patterns. **a** The enrichment of TFBSs was different between TSSs with different H3K4me3 patterns. The enrichment was spread across a broader region at TSSs with non-narrow H3K4me3 peaks, suggesting that TF binding plays a role in maintaining H3K4me3 breadth. Matches to 2414 known protein-binding motifs were searched within the −1 to 1 kb region around TSSs. We found that 340 motifs were enriched by at least 20 % at TSS with H3K4me3. The average enrichment of these motifs was plotted separately for each H3K4me3 pattern. **b** The UP00408 motif of GABPA was highly enriched at TSSs with narrow H3K4me3 peaks

We matched DNA sequences around TSS to 2414 known protein-binding motifs to identify potential transcription factor binding sites (TFBSs) and found 340 motifs enriched around TSSs with H3K4me3 in primary monocyte. The enrichment of these motifs corresponded to H3K4me3 breadth at TSSs (Fig. 6a). There is significant overlap between the lists. We used IPA to determine cellular functions associated with each set of TFBSs. The broad symmetric peak pattern was associated with TFBSs that regulate cell death (*p* = 9.6 × 10^−13^), differentiation of cells (*p* = 1.0 × 10^−9^), and organismal development (*p* = 3.3 × 10^−8^). Network analysis showed strong nodes of PI3K, STAT1, and NFκB. The narrow peak was associated with TFBSs that regulate cell hyperplasia (*p* = 3.8 × 10^−8^) and cell differentiation (*p* = 6.6 × 10^−7^). Network analysis showed strong nodes of ERK, EGR, NFκB, AKT, and JNK. The upstream extended peak pattern was associated with TFBSs that regulate exit from the cell cycle (*p* = 3.2 × 10^−8^) and had network nodes comparable to that of the narrow peak. The downstream extended peak pattern was associated with TFBSs that regulate cell death (*p* = 8.6 × 10^−11^), exit from the cell cycle (*p* = 2.6 × 10^−10^), G1 phase (*p* = 4.5 × 10^−10^), arrest in G1 phase (*p* = 1.4 × 10^−8^), entry into S phase (*p* = 2.2 × 10^−8^), and colony formation (*p* = 2.2 × 10^−8^). Network analysis showed strong nodes of E2F1, cyclin A, and SP1. As an example, GA binding protein transcription factor alpha subunit (GABPA) regulates myeloid and lymphoid cell-specific genes and is required for innate immunity as well as proliferation of fibroblasts [18, 19]. Its potential binding sites are highly enriched around TSSs with the narrow peak H3K4me3 only (Fig. 6b).

### Generalization of the H3K4me3 breadth-transcription association in inflammation

**Fig. 7:**
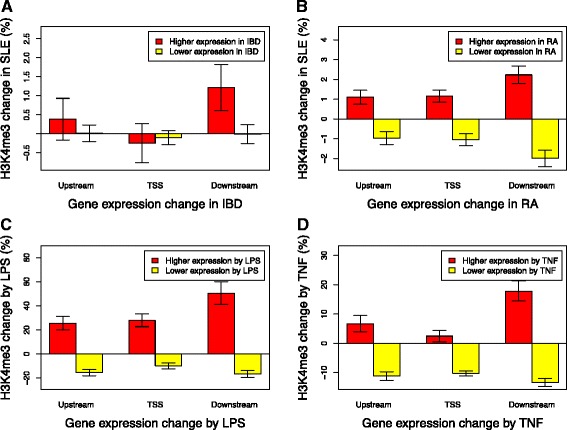
Validation in other inflammatory models. **a** Genes with significantly increased or decreased transcription in inflammatory bowel disease (IBD) were obtained from our unpublished RNA-seq data. The average H3K4me3 changes in our unmatched SLE samples were calculated at three different regions, showing that the only significant change was the increase of downstream H3K4me3 at TSS of genes with increased transcription. **b** The same analysis was applied to a published data set (GSE10500) comparing the transcriptome of rheumatoid arthritis (RA) patients and controls in macrophage. **c** This analysis used matching RNA-seq and H3K4me3 data obtained from primary monocytes treated with LPS or cell culture medium as control (GSE58310). Average H3K4me3 changes of gene sets with increased or decreased transcription by LPS were calculated for the same three regions. *Error bars* indicate that average H3K4me3 was changed significantly towards the same direction as the change of transcription. **d** The same analysis was applied to matching ChIP-seq H3K4me3 and microarray transcriptome data generated from TNF-treated endothelium (GSE54000). All plots normalized H3K4me3 data to make the average change of all genes equal to zero at each region

We performed several analyses to test whether the association between differential gene transcription and downstream H3K4me3 is unique to SLE monocytes. The first analysis used our unpublished RNA-seq data obtained from whole blood samples of inflammatory bowel disease (IBD). There were, respectively, 136 and 472 genes with significantly increased and decreased transcription in IBD patients comparing to unaffected controls. Since the matching H3K4me3 data from the same samples is not available, data from our SLE samples was used to calculate the average H3K4me3 change of these genes at three different regions (Fig. 7a). Change in downstream H3K4me3 in SLE patient was associated with higher expression in IBD (*p* = 0.046). The same analysis was applied to a published microarray data set (GSE10500) comparing the CD14+ macrophage transcriptome of rheumatoid arthritis (RA) patients and control samples. Correlation between transcription change in RA and H3K4me3 change in SLE existed at all three regions but was the strongest at TSS (Fig. 7b). The transcription-H3K4me3 correlation was stronger for RA than for IBD, probably because of the difference in cell type. Therefore, despite of the remarkably different cell and disease types of these data sets, integrative analysis of multiple diseases suggested that the concordant increase of downstream H3K4me3 and transcription occurs commonly in blood cells of inflammatory diseases.

Several published data sets included matching transcriptome and H3K4me3 data from experiments that induced inflammatory responses in human cells. We downloaded two of these data sets from Gene Expression Omnibus and re-analyzed them using the same method as above. The GSE58310 data set included RNA-seq data from macrophages treated with or without LPS and matching ChIP-seq H3K4me3 data [20]. To emphasize an epigenetic effect, the RNA-seq data was collected after LPS washout and cell culture for six more days. Genes maintaining differential transcription after the washout were identified from the RNA-seq and their average H3K4me3 changes were calculated at different regions (Fig. 7c). The other data set (GSE54000) included microarray transcriptome data from endothelium treated with TNF and matching ChIP-seq H3K4me3 data [21]. The same analysis was applied to genes whose transcription was significantly modified by TNF (Fig. 7d). LPS and TNF are both inducers of inflammatory responses and our recent study reported elevated LPS level in circulating blood of SLE patients [11]. In both cases, concordant changes of transcription and H3K4me3 were observed at all three regions around TSS; however, the largest H3K4me3 change always happened at TSS downstream region of genes with increased transcription. These analyses validate our observation in SLE and support a general theme of downstream H3K4me3 dictating capacity for stimulus-inducible transcription.

## Conclusions

H3K4me3 breadth has recently been identified as a key regulator of cell type identity [5]. Very broad domains of H3K4me3 were identified as lineage specific markers in both human and mouse. These broad domains were typically extended over 5 kb and highly remodeled during cell differentiation. Enrichment of lineage specific transcription factors within these broad domains was observed, thereby supporting the concept that the domains were critically associated with lineage commitment. The H3K4me3 mark is deposited by members of the COMPASS/Trithorax family of methyltransferases and is removed by the JARID family of demethylases [22]. The H3K4me3 marked nucleosome can be dynamically acetylated by p300 and CBP [23]. This subsequent step may represent a mechanism by which H3K4me3 regulates transcription. We wished to understand the effects of H3K4me3 breadth at TSSs, a subject that had not been previously addressed. We had already identified significant changes in H3K4me3 peak height in the setting of SLE. This study was undertaken specifically to evaluate effects of H3K4me3 peak breadth in a human disease state.

We found that TSS H3K4me3 patterns were not markedly changed in SLE although monocyte behavior is markedly changed in SLE [12, 24–26], and transcription changes underlying the altered behavior are also substantial [11, 14–16]. It was surprising, therefore, to find that the H3K4me3 patterns themselves were largely stable in SLE monocytes.

We noted that the TSS and the downstream H3K4me3 changes were most closely aligned with differential transcription in SLE. Upstream changes in H3K4me3 were not directly associated with differential transcription once the data were corrected for dependence of adjacent regions on each other. The downstream extended category of H3K4me3 was the pattern most strongly associated with inflammation and immune responses. It is then expected that this would be the set of genes most altered in the setting of SLE. The nucleosome downstream of the TSS is important functionally. H3K4me1 tends to locate next to the outer edge of H3K4me3 (Fig. 1c), so its peak breadth is correlated with H3K4me3 peak breadth. H3K4me1 is required for the recruitment of factors that interact with H3K4me3 [27]. This association may therefore relate to restriction of the activities nucleated on the downstream nucleosome. Many TSSs in this study had downstream H3K4me3 extended to ~650 bp, where H3K36me3, a transcriptional elongation mark, starts to increase (Fig. 1c). Release of RNA polymerase from pausing occurs at this location, and histone acetylation of this downstream nucleosome appears to be central to the process and is at least partly dependent on H3K4me3 [23, 28–30]. Therefore, modifications at this downstream nucleosome may control pivotal events in transcriptional elongation. These findings also have important implications for the analysis of ChIP-seq data that typically focuses on the nucleosome upstream of the TSS. These data highlight the importance of a comprehensive assessment of changes in analytic approaches.

This conclusion is seemingly contradictory to what was described in the seminal paper on H3K4me3 breadth [5]. That paper reported the effect of H3K4me3 breadth on cell identity by comparing different cell types while the current study was focused on one cell type under different pathological states. Another source of the different conclusions between the two studies is the difference in the definition of broad H3K4me3 peaks. In that paper, the broad H3K4me3 domains spanned up to 60 kb and had minimal length of over 4 kb in hESC. The domains were largely intergenic. This study, on the other hand, only looked at a much narrower region of 1 kb around TSSs, which might have more direct association with transcription level and variability. The breadth of narrow and broad peaks usually differed only by hundreds of base pairs.

This study makes unique contributions by defining H3K4me3 patterns at TSSs and by identifying the nucleosome downstream of the TSS as directly associated with transcription. Given that many genes have the transcriptional initiation-elongation transition in this region [31, 32], it is plausible to hypothesize that increase of downstream H3K4me3 will facilitate the transition by making the nucleosome more accessible to elongation machinery. Nevertheless, the study has some important limitations. The sample size of the SLE patients was relatively small and included patients with mild or moderate disease activity. As higher levels of disease activity could recruit additional gene expression changes as well as H3K4me3 changes, our study may underestimate the effects. An additional limitation was the focus on the annotated TSS region. We chose to focus on the TSS in order to link our findings with our RNA-seq data.

In summary, our study highlights the importance of examining H3K4me3 breadth patterns as well as peak height in evaluating ChIP-seq data. This is also one of the first studies to examine the changes in H3K4me3 patterns related to a disease state. Furthermore, data mining analyses of extra data sets further suggested that the association between transcription and downstream H3K4me3 is common to inflammatory responses. Our results emphasize the stability of the patterns and the importance of the downstream nucleosome in regulating gene transcription.

## Methods

Primary monocytes from six SLE patients with mild to moderate disease activity and six unrelated controls were isolated as described [33]. The primary data on H3K4me3 and RNA-seq have been previously published [11, 34]. The SLEDAI scores on the day of sampling were between 0 and 7 (mean = 2.50). Detailed sample description was given in Supplemental Table 1 of [11]. Chromatin immunoprecipitation of H3K4me3 was carried out as previously described [35–38]. Immunoprecipitation with anti-GST (Invitrogen, Camarillo, CA) and input were used to define background. The library preparation utilized the SOLiD ChIP-seq kit and was performed according to the manufacturer’s instructions. The same samples had parallel RNA-seq data, which was previously reported [11].

We refined our previously described “CHOP-seq” pipeline to process the ChIP-seq data [39]. After the sequencing reads were mapped to human reference genome (hg19), all reads were extended to 200 bp long at the 3′ end to cover the isolated region of nucleosome occupancy. The sequencing depth around 27,588 uniquely located transcription start sites (TSSs) annotated by RefSeq was summarized to obtain a measurement for each TSS and sample. A normalized measurement of −1.0 and 1.0, respectively, corresponds to average depth two times lower and higher than the background. The measurements were first normalized between samples and then adjusted by subtracting background signal measured by input controls. The detail of these steps was described in our previous paper [33].

We started with the H3K4me3 at 27,588 annotated TSSs of six control samples and then related the H3H4me3 patterns identified from these samples to relative H3K4me3 changes in six SLE samples. The normalized depth at single bases in the −1 to 1 kb region around TSS was averaged into 50 bp bins to obtain two 27,588 × 41 matrixes for the control and SLE group, respectively. The EM algorithm for mixtures of univariate normals method (https://cran.r-project.org/web/packages/mixtools) was applied to three different regions (TSS, 400–500 bp upstream, and 600–700 bp downstream) to identify patterns of H3K4me3 breadth, using only TSSs with substantially higher H3K4me3 level than background. To analyze gene set overrepresentation of each pattern, we collected predefined gene sets from various sources such as BioSystems, KEGG, and OMIM. The hypergeometric test was applied to each gene set to identify those significantly enriched within genes having a specific H3K4me3 breadth pattern. All genes having detectable H3K4me3 at their TSS were used as test background.

RNA-seq results from the same samples were obtained from our previous study [11]. Genes with significant differential transcription between control and SLE samples (*p* < 0.01) were used to establish associations between differential H3K4me3 and differential transcription. Genes with multiple TSSs classified into different H3K4me3 patterns were not included. RNA-seq reads from whole blood of three patients with very early-onset inflammatory bowel disease and their six unaffected parents were aligned to the reference genome GRCh37 using Tophat2. Transcriptome assembly was performed using Cufflinks, after which the assemblies were merged across samples using Cuffmerge. Differentially expressed genes between the probands and their parents were identified using Cuffdiff (FDR < 0.05). The publication of this data set is in preparation.

Extra ChIP-seq data of CTCF and 11 histone modifications in CD14+ monocyte, such as H3K27ac and H3K36me3, were obtained from ENCODE (https://genome.ucsc.edu/ENCODE). These data were processed in the same way to get a 27,588 × 41 matrix for each histone modification. A combined set of 2414 protein-binding DNA motifs was collected from public resources, such as TRANSFAC and ENCODE. Matches to these motifs were searched for within −1 to +1 kb of all TSSs and considered as potential transcription factor binding sites (TFBSs). Enrichment and distribution of matches were compared across TSS subgroups with different H3K4me3 patterns. We re-analyzed two published data sets including matching H3K4me3 and transcriptome samples treated with TNF or LPS. Processed data of both data sets (GSE 54000 and GSE58310) were downloaded from the Gene Expression Omnibus (http://www.ncbi.nlm.nih.gov/geo).

Source code of all data analysis is publically available at https://goo.gl/22csxb. A cloud-based interactive online tool (http://awsomics.org/project/sle_h3k4me3_breadth) was created to share the data and results of this study. This tool integrates the data of this project with a variety of public genomic data we are continuously collecting from public repositories through Amazon Web Service. It allows users to explore, query, and visualize the data in an integrative way.

## Availability of supporting data

We designed an innovative way for research to share their genomic data and presented it for the first time through this study. An online tool was implemented and made publicly available (http://awsomics.org/project/sle_h3k4me3_breadth) for users to access data of this study and perform exploratory analysis and customized visualization themselves (Additional file 3: Figure S3 and Additional file 4: Figure S4). This tool was built upon a cloud-based framework and integrated into a variety of collection of genomic information, such as predefined gene sets and protein-binding motifs used in this study.

## Additional files

Additional file 1: Figure S1. Bimodal distribution of TSS H3K4me3 and difference of H3K4me3 levels between nearby regions. A) The distribution of H3K4me3 sequencing depth followed a bimodal distribution. The left mode corresponds to background noise from TSSs without H3K4me3 while the right mode corresponds to different levels of H3K4me3 at the other TSSs. We selected the 14,217 TSSs classified into the right mode with high confidence for further analysis. B) The difference of H3K4me3 between TSS and upstream/downstream regions also followed bimodal distribution. The combination of these two distributions defined four H3K4me3 patterns. For example, TSSs classified into the left mode of both distributions had the narrow peak pattern while those classified into both right modes had the broad symmetric pattern as their H3K4me3 level at both upstream and downstream regions was similar to that at TSS. Additional file 2: Figure S2. ENCODE data showing histone modifications. Many other histone modifications had patterns complementing to four distinctive H3K4me3 patterns. Sequencing depth from ChIP-seq data of CTCF and 11 histone modifications in CD14+ monocyte was obtained from the ENCODE project. Additional file 3: Figure S3. A snapshot of our online tool to support the accessibility of genomic data used in this study. Users can pick a gene or gene set to plot its H3K4me3 landscape around TSS while a large collection of gene sets is available for selection through the tool. This plot shows the average control-patient difference of seven genes of “Innate immune response activating cell surface receptor signaling pathway” (GO:0002220). Average increase of H3K4me3 can be seen at upstream region with small peaks about 200 bp in size, likely corresponding to individual nucleosome units. Additional file 4: Figure S4. Snapshot of a 15-bp motif of IRF1 binding. This snapshot from the same tool shows that a 15-bp motif of IRF1 binding is enriched at TSSs with broad H3K4me3 peaks but not at TSSs with narrow peaks. Users can access a collection of over 2400 motifs through this tool.

## Abbreviations

H3K4me3: tri-methylation of histone H3 lysine
SLE: systemic lupus erythematosus
TFBS: transcription factor binding site
TSS: transcription start site
