# Supplementary figures and images for: H3K4 tri-methylation breadth at transcription start sites impacts the transcriptome of systemic lupus erythematosus
Source: Clin Epigenetics. 2016 Feb 2;8:14. doi: 10.1186/s13148-016-0179-4 (PMC4736279; doi:10.1186/s13148-016-0179-4)

# **A** Bimodal distribution of TSS H3K4me3

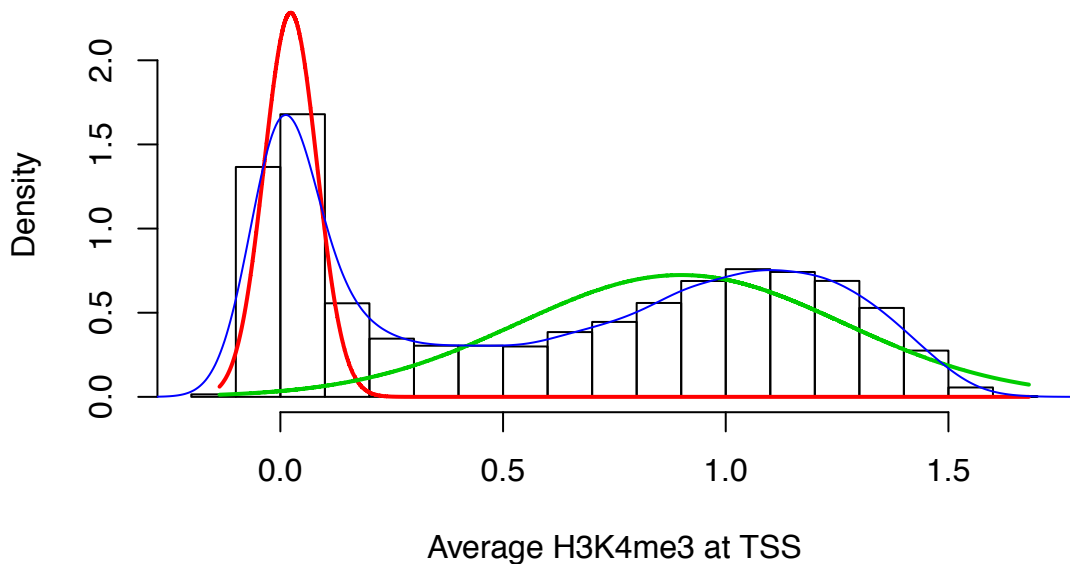

# **B** Difference of H3K4me3 levels between nearby regions

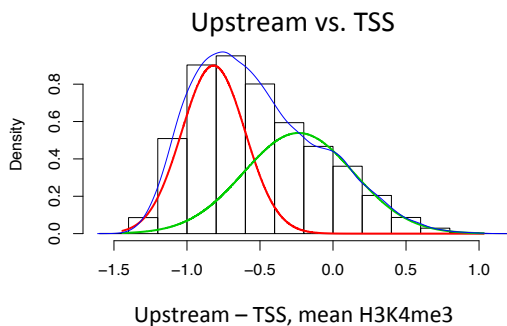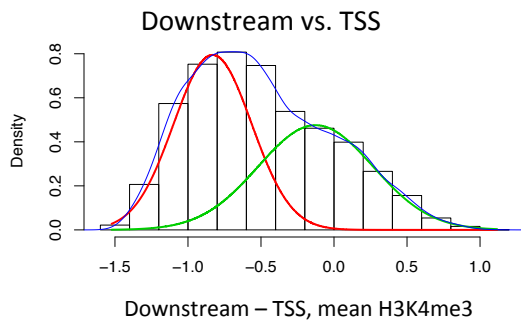

Supplement: Additional file 1: Figure S1. — Bimodal distribution of TSS H3K4me3 and difference of H3K4me3 levels between nearby regions. A) The distribution of H3K4me3 sequencing depth followed a bimodal distribution. The left mode corresponds to background noise from TSSs without H3K4me3 while the right mode corresponds to different levels of H3K4me3 at the other TSSs. We selected the 14,217 TSSs classified into the right mode with high confidence for further analysis. B) The difference of H3K4me3 between TSS and upstream/downstream regions also followed bimodal distribution. The combination of these two distributions defined four H3K4me3 patterns. For example, TSSs classified into the left mode of both distributions had the narrow peak pattern while those classified into both right modes had the broad symmetric pattern as their H3K4me3 level at both upstream and downstream regions was similar to that at TSS. [file 13148_2016_179_MOESM1_ESM.pdf]
