# Supplementary material for: H3K4 tri-methylation breadth at transcription start sites impacts the transcriptome of systemic lupus erythematosus
Source: Clin Epigenetics. 2016 Feb 2;8:14. doi: 10.1186/s13148-016-0179-4 (PMC4736279; doi:10.1186/s13148-016-0179-4)
Supplement: Additional file 2: Figure S2. — ENCODE data showing histone modifications. Many other histone modifications had patterns complementing to four distinctive H3K4me3 patterns. Sequencing depth from ChIP-seq data of CTCF and 11 histone modifications in CD14+ monocyte was obtained from the ENCODE project. [file 13148_2016_179_MOESM2_ESM.pdf]

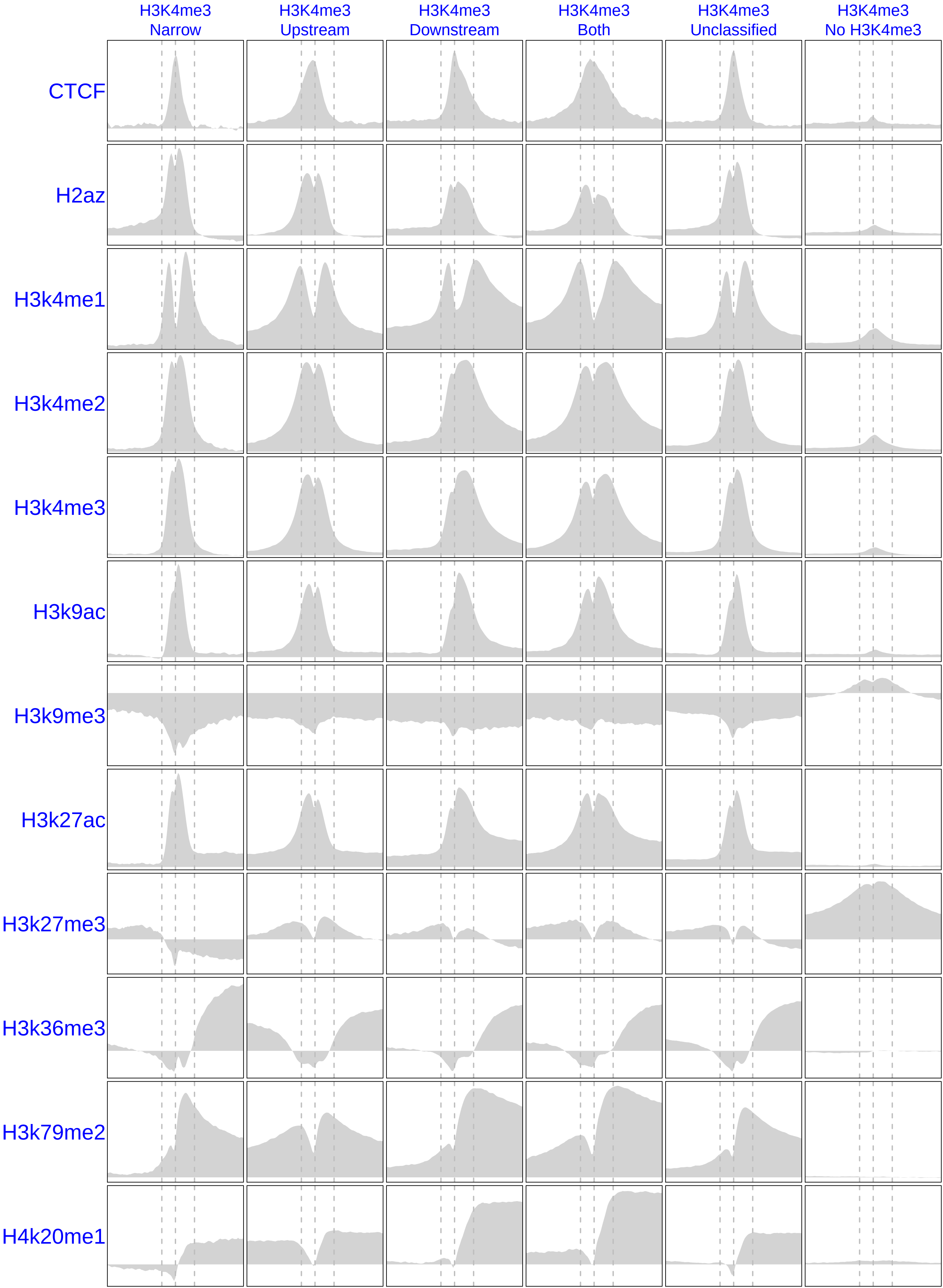
H3K4me3 Narrow
H3K4me3 Upstream
H3K4me3 Downstream
H3K4me3 Both
H3K4me3 Unclassified
H3K4me3 No H3K4me3
CTCF
H2az
H3k4me1
H3k4me2
H3k4me3
H3k9ac
H3k9me3
H3k27ac
H3k27me3
H3k36me3
H3k79me2
H4k20me1
