# Supplementary material for: H3K4 tri-methylation breadth at transcription start sites impacts the transcriptome of systemic lupus erythematosus
Source: Clin Epigenetics. 2016 Feb 2;8:14. doi: 10.1186/s13148-016-0179-4 (PMC4736279; doi:10.1186/s13148-016-0179-4)

H3K4me3 breadth | Home | Data | Result | Visualization

- H3K4me3 sequencing depth
- Expression-H3K4me3 overlapping
- TFBS frequency

Plot H3K4me4 peaks

Plot sequencing depth of H3K4me3 at one or a set of TSSs. The depth was the normalized average of 6 control or 6 SLE samples. By default, the plotted data is in log2 scale, so a difference of 1.0 is the same as 2 fold change. Plot options:

- "Plot unit": plot individual TSS or all TSSs mapped to a gene set.
- "Plot value": plot control and SLE data separately, or their difference (SLE - control).
- "Plot type": plot type (landscape vs. barplot).
- "Plot scale": plot data in log2 scale (default), or unlogged scale.
- "Plot range": plot range around TSS (-10kb to +10kb).

Select a TSS

Search:

ID	Gene	Gene_ID	Chr	Location	Strand	H3K4me3_Control	H3K4me3_SLE
chr1_1243269	ACAP3	116983	chr1	1243269	-	Both	Both
chr1_1709909	NADK	65220	chr1	1709909	-	Both	Both
chr1_1710297	NADK	65220	chr1	1710297	-	Both	Both
chr1_1822526	GNB1	2782	chr1	1822526	-	Both	Both
chr1_6052357	KCNAB2	8514	chr1	6052357	+	Both	Both
All	A	All	.	All	All	All	All

Showing 1 to 5 of 27,588 entries

Previous Next

Select a gene set

BioSystems | GO | Clear selection

Search: immune

ID	Name	Size
GO:0002200_human	somatic diversification of immune receptors	47
GO:0002204_human	somatic recombination of immunoglobulin genes involved in immune response	18
GO:0002208_human	somatic diversification of immunoglobulins involved in immune response	18
GO:0002218_human	activation of innate immune response	156
GO:0002220_human	innate immune response activating cell surface receptor signaling pathway	7

Showing 1 to 5 of 150 entries (filtered from 19,440 total entries)

Previous Next

Select plot unit
- Individual TSS
- Gene set average

Select plot value
- Control/SLE separately
- Control-SLE difference

Select plot type
- Plot landscape
- Plot bar

Select plot scale
- Logged
- Unlogged

Select plot range

-10,000 | -2,000 | 2,000 | 10,000

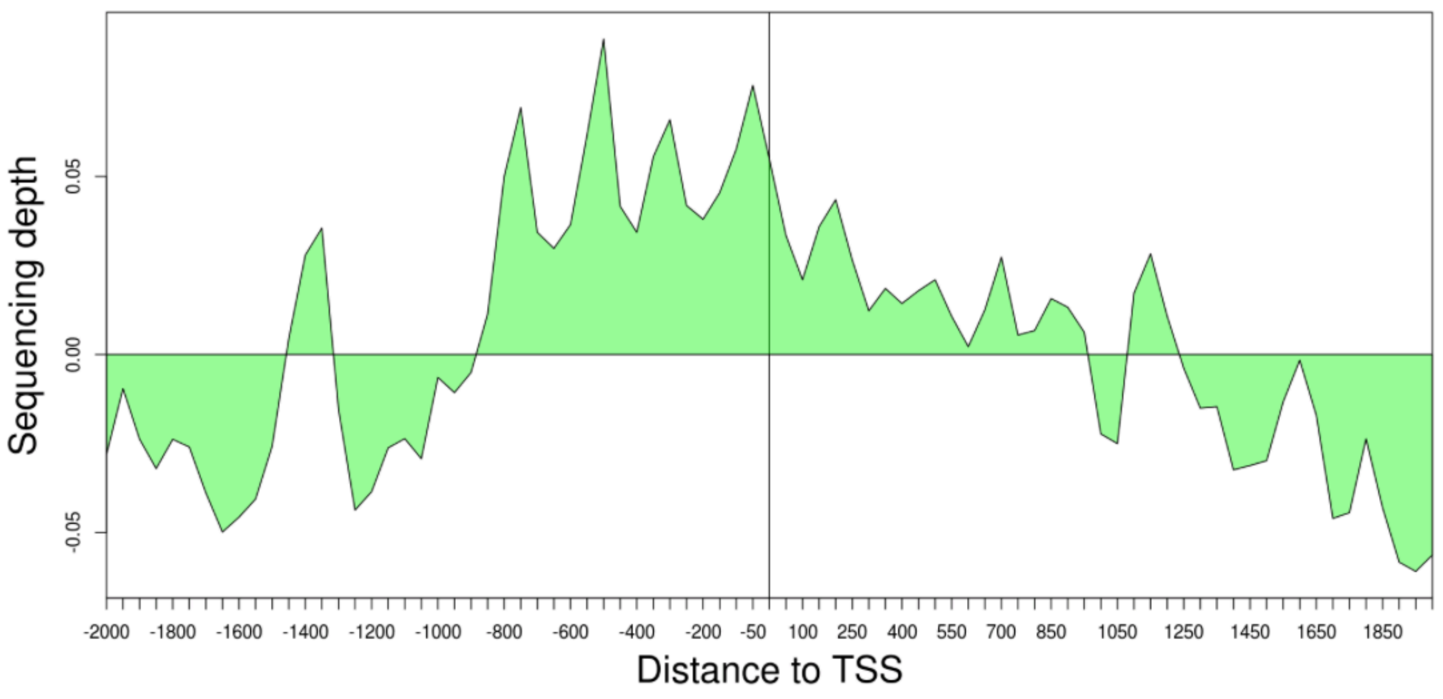

Supplement: Additional file 3: Figure S3. — A snapshot of our online tool to support the accessibility of genomic data used in this study. Users can pick a gene or gene set to plot its H3K4me3 landscape around TSS while a large collection of gene sets is available for selection through the tool. This plot shows the average control-patient difference of seven genes of “Innate immune response activating cell surface receptor signaling pathway” (GO:0002220). Average increase of H3K4me3 can be seen at upstream region with small peaks about 200 bp in size, likely corresponding to individual nucleosome units. [file 13148_2016_179_MOESM3_ESM.pdf]
