# Supplementary material for: H3K4 tri-methylation breadth at transcription start sites impacts the transcriptome of systemic lupus erythematosus
Source: Clin Epigenetics. 2016 Feb 2;8:14. doi: 10.1186/s13148-016-0179-4 (PMC4736279; doi:10.1186/s13148-016-0179-4)

Plot TFBS frequency

Plot frequency of matches to each PWM (point weighted matrix) at TSS. TSSs were grouped based on H3K4me3 peak breadth. Plot options:

- "Select H3K4me3 pattern": H3K4me3 peak patterns, means no detectable H3K4me3 higher than background.
- "Plot relative enrichment": Plot relative enrichment to if checked.

Select H3K4me3 pattern

- [x] Narrow
- [ ] Upstream
- [ ] Downstream
- [x] Both
- [ ] Unclassified
- [x] No H3K4me3

- [x] Plot relative enrichment

Select a PWM

Search:

ID	Name	Length	Source	Consensus	GC	N	Narrow	Upstream	Downstream	Both	Unclassified	No.H3K4me3
EN0200	IRF1	15	ENCODE	YYRRCCAATCRRARS	0.4945534	27509	0.9560	1.2484	1.0445	1.7856	1.1590	1
EN0201	IRF1	10	ENCODE	RAAASYGAAA	0.2831776	83421	1.1348	0.7805	0.7400	0.4702	0.8338	1
EN0202	IRF1	15	ENCODE	TGASTCATWWYGRRA	0.3927778	31152	1.1352	0.6353	0.6157	0.3363	0.6929	1
EN0203	IRF1	10	ENCODE	RRGGAASTGR	0.5330189	117666	0.9426	1.0530	1.0709	0.9764	1.0420	1
EN0204	IRF1	9	ENCODE	AGTCATYTC	0.3956638	36835	1.0411	0.6903	0.6884	0.4338	0.7795	1
				All				Al	All		All	All

Showing 1 to 5 of 17 entries (filtered from 2,414 total entries)

Previous Next

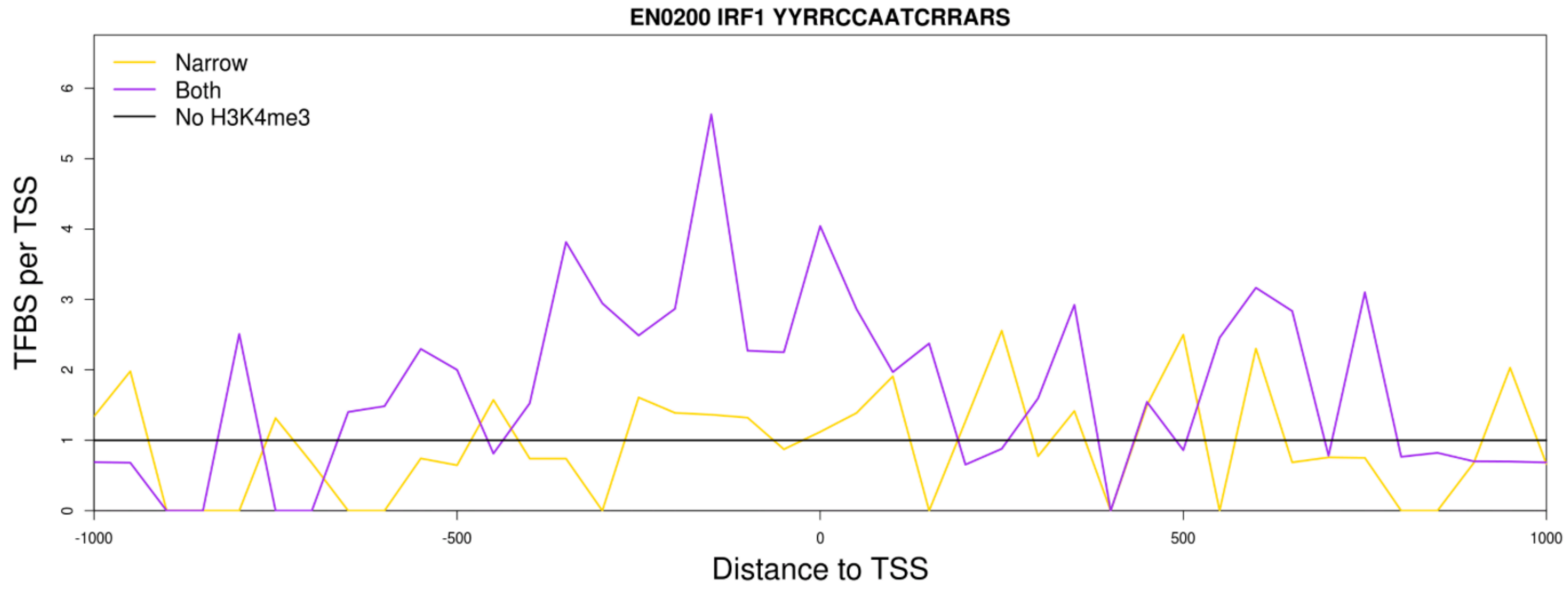

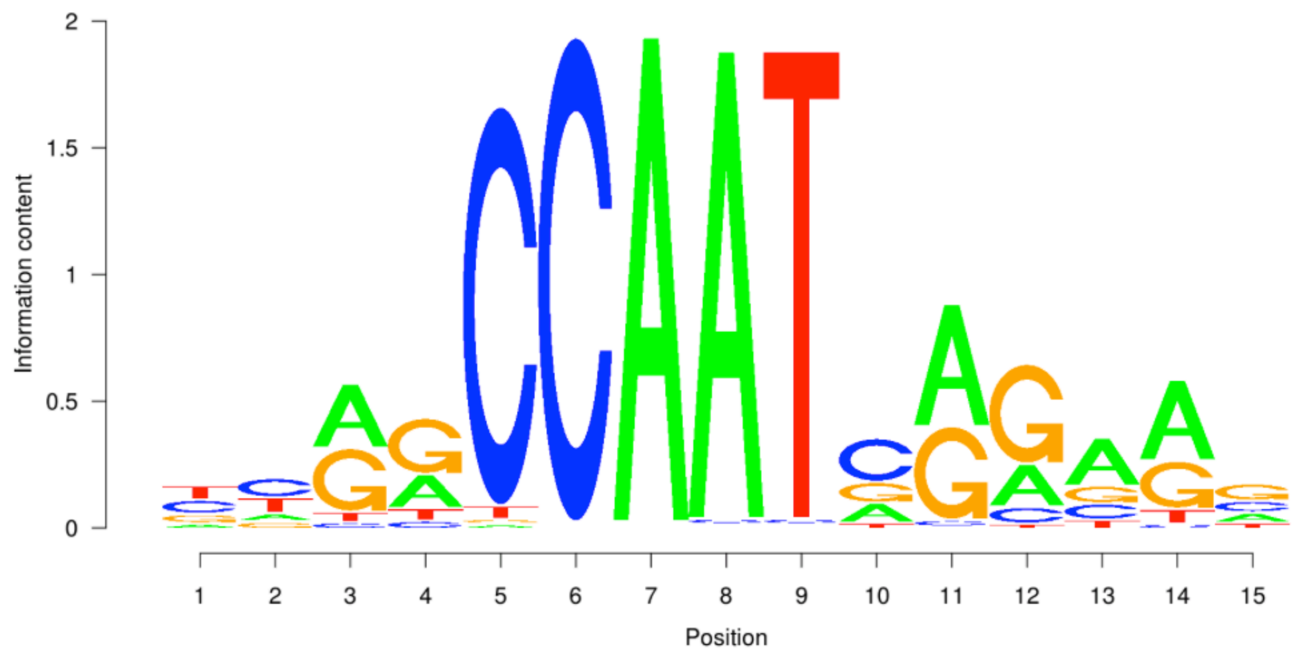

Supplement: Additional file 4: Figure S4. — Snapshot of a 15-bp motif of IRF1 binding. This snapshot from the same tool shows that a 15-bp motif of IRF1 binding is enriched at TSSs with broad H3K4me3 peaks but not at TSSs with narrow peaks. Users can access a collection of over 2400 motifs through this tool. [file 13148_2016_179_MOESM4_ESM.pdf]
